# Mixed Adenoneuroendocrine Cancer of the Duodenum Causing Gastric Outlet Obstruction

**DOI:** 10.14309/crj.0000000000000787

**Published:** 2022-06-23

**Authors:** Aimen Farooq, James Wert, Baha Aldeen Bani Fawwaz, Abu Hurairah

**Affiliations:** 1 Department of Internal Medicine, AdventHealth Orlando, Orlando, FL; 2 Division of Gastroenterology, Department of Internal Medicine, AdventHealth Orlando, Orlando, FL

## CASE REPORT

**Figure 1.**
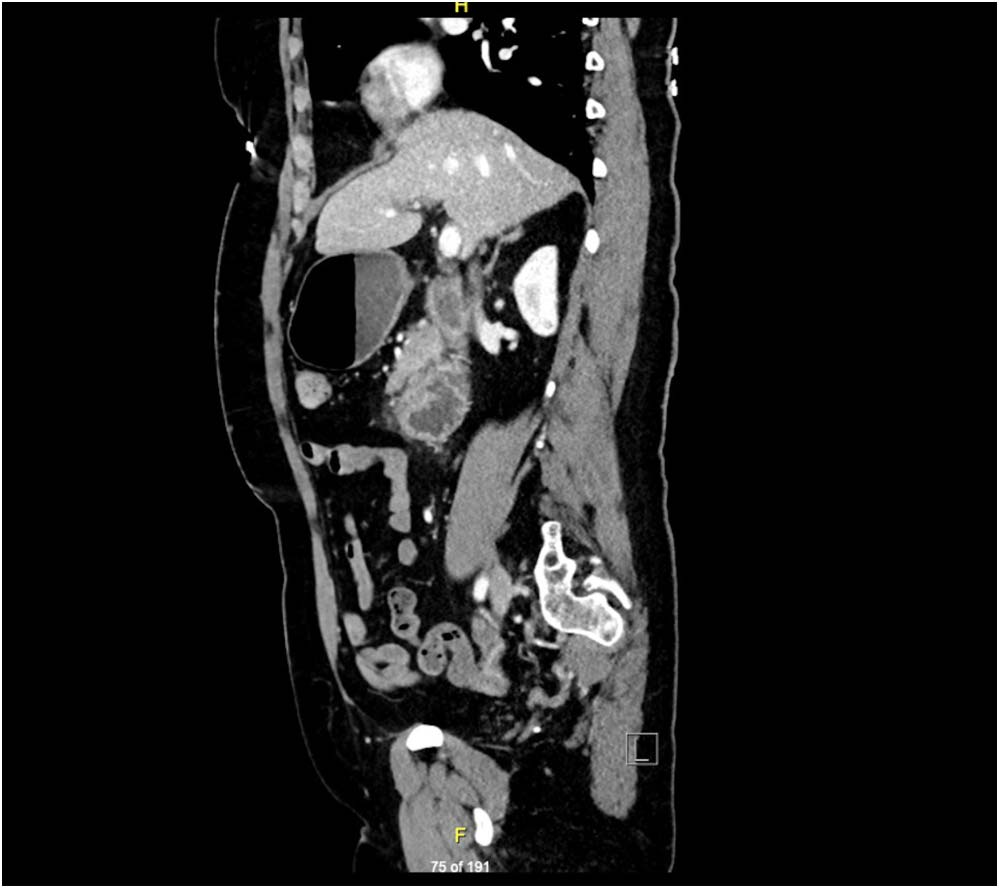
Sagittal view of a contrast-enhanced computed tomography scan of the abdomen showing stomach distension and a thickened, narrow segment of the third portion of the duodenum with surrounding enlarged lymph nodes.

**Figure 2.**
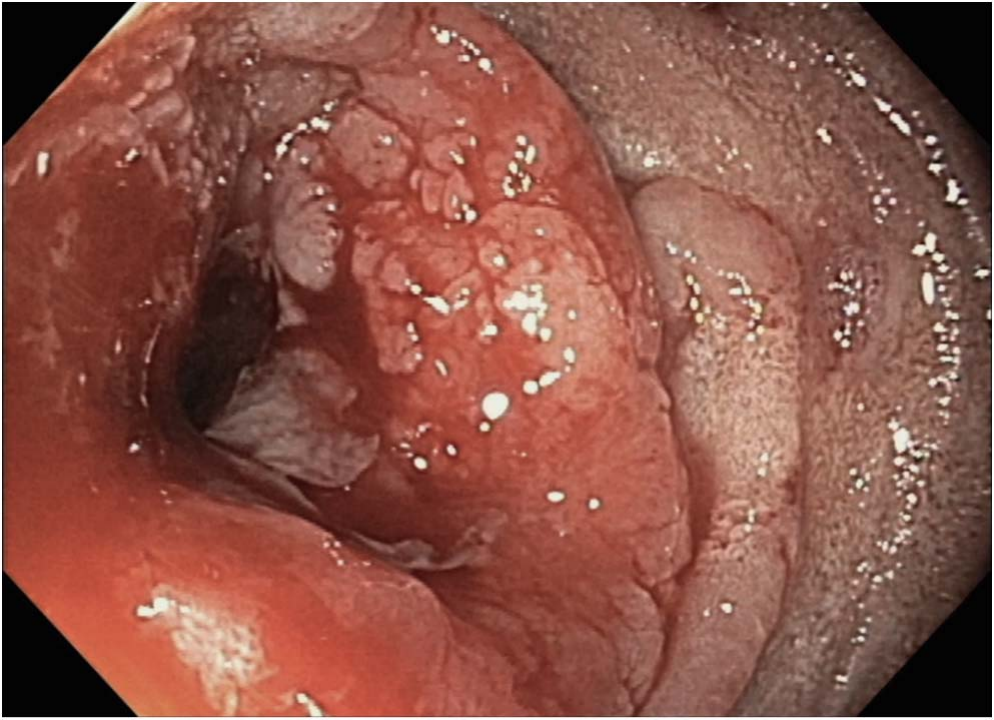
An infiltrating mass in the third portion of the duodenum as seen during endoscopy.

**Figure 3.**
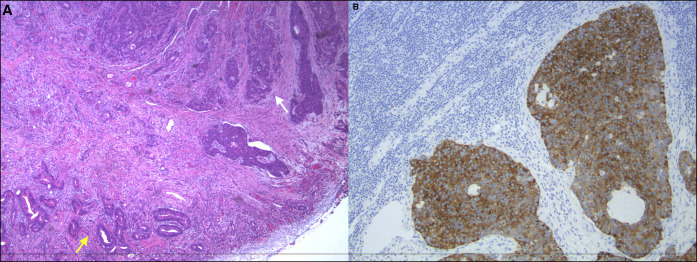
(A) The primary tumor showing a gland forming an adenocarcinoma component along the left (yellow arrow) and a high-grade neuroendocrine (NEC) cancer along the upper right (white arrow). NEC component has coarse “salt and pepper” nuclear chromatin and frequent mitoses. (B) Lymph node metastasis with immunohistochemical stains showing the diffuse expression of synaptophysin, a NEC marker.

A 57-year-old woman with a medical history of diverticulitis and small bowel obstruction presented with nausea, vomiting, epigastric pain, and weight loss. Her initial examination was unremarkable except mild epigastric tenderness. Abdominal computed tomography showed a distended stomach with a thickened and narrow third portion of the duodenum (Figure 1). Esophagogastroduodenoscopy revealed a bleeding mass in the duodenum with pathology confirming moderately differentiated adenocarcinoma (Figure 2). A subtotal pancreaticoduodenectomy was performed, and surgical pathology revealed poorly differentiated mixed adenocarcinoma-neuroendocrine cancer forming a 3.2 cm mass invading through the duodenal wall into the adjacent pancreas, peripancreatic tissue, and regional lymph nodes (Figure 3). The Ki67 proliferation index was approximately 70%, and synaptophysin was positive (Figure 3). The tumor stage was IIIC, T4aN2M0 requiring adjuvant FOLFOX chemotherapy. Unfortunately, she was unable to tolerate chemotherapy because of the side effects. Surveillance computed tomography of the abdomen revealed new hepatic lesions. Pembrolizumab was started given the high tumor mutational burden, with yet still progression of disease. The patient continues pembrolizumab while awaiting a study trial.

Mixed adenoneuroendocrine cancer of the digestive system is a rare entity consisting of both adenocarcinomatous and neuroendocrine (NEC) differentiation, each component representing at least 30%.^1^ This presents a diagnostic dilemma when it comes to preoperative diagnosis based on endoscopic biopsies because adenocarcinoma is the main intramucosal component. Moreover, NEC may exhibit microscopic patterns like poorly differentiated or undifferentiated adenocarcinoma.^1^ A detailed microscopic and immunological identification of the neuroendocrine component and the quantitative assessment of the Ki67 index are essential to make accurate diagnosis and determine an effective therapeutic strategy for these aggressive tumors with a median overall survival of 13.2 months.^2^

Surgical resection is the only curative option for resectable lesions per the National Comprehensive Cancer Network guidelines; however, postoperative adjuvant therapy is usually required to prevent recurrence. The National Comprehensive Cancer Network recommends a platinum-based regimen to target the NEC.^3^ Apostolidis et al.^4^ demonstrated no significant difference between platinum and etoposide or oxaliplatin and 5‐fluorouracil‐based regimens as first‐line therapy because NEC shares a genetic profile with conventional colorectal cancers. There are emerging research studies where immune checkpoint therapy is showing response in high tumor mutational burden expression.^5^ The most effective regimen for mixed adenoneuroendocrine cancer, however, remains unclear.

A host of other tumor markers and genes (KRAS G12V, APC, ARID2, ERBB3, and p53 mutations) were detected by molecular profiling with unclear clinical significance. Nonetheless, the importance of detecting these mutations cannot be understated because they may 1 day become potential targets for novel therapies.

## DISCLOSURES

Author contributions: All authors mentioned above contributed to the conception, design, drafting, finalizing, and approving the final article in order of appearance and meet the ICJME criteria for authorship with the first author (A. Farooq) being the main contributor. All authors are willing to accept accountabilities for all aspects of the article. BAB Fawwaz worked with A. Farooq on gathering radiology and pathology images. A. Hurairah also served as a mentor during the preparation of this article.

Financial disclosure: None to report.

Previous presentations: This case has been presented in the 2021 Annual Southern Medical Association meeting in Lake Buena Vista, FL, on October 28, 2021, where the presentation was awarded a third prize.

Informed consent was obtained for this case report.
